# Fatigue as a Cause of Consumption

**Published:** 1902-04-12

**Authors:** 


### Hospital Clinics and Medical Progress.

### FATIGUE AS A CAUSE OF CONSUMPTION.

Writing on the etiology of pulmonary tuberculosis, Dr. Burton-Fanning [1] draws attention afresh to the long time which may elapse between the reception of the infection and the manifestation of symptoms of such gravity as to suggest the existence of tuberculosis. It is usual, he says, for an indefinite length of time to elapse between the lodgment of the tubercle bacillus in the body and the manifestation of symptoms of consumption. In some cases nothing may have occurred to show, even in the light of subsequent events, that the patient was previously the subject of tuberculous deposit. In the larger number, however, we may elicit by questioning, clear evidence of the existence of tuberculosis prior to the date which the patient gives as that of the commencement of his illness.

Thus we may explain and reconcile the long-standing difference between pathologists and clinicians in regard to the infectivity of the disease. The clinical histories of many patients point to the fact, Dr. Burton-Fanning says, that early in their life the tubercle bacillus gained an entrance to their bodies. It then secreted itself in a lymphatic gland or portion of lung, and lay dormant for any number of years. Thus the individual who eventually develops pulmonary tuberculosis has usually been harbouring the virus of the malady for a number of years, and the definite onset of symptoms only marks his inability to hem in the bacillus any longer. According to this conception of the sequence of events, although, pathologically, the bacillus retains its place as the *causa causans* of the disease; clinically, the implantation of the infection fades away into bygone history, and assumes the position of the implantation of a "tendency," and so far as the existing condition is concerned, the exciting cause of the present malady has to be sought among those accidents which have allowed this "tendency" to manifest itself, or in more accurate language, have enabled the long dormant tuberculosis to burst its bounds and infect new areas of tissue. This is a view which, if thoughtfully worked out, must have far-reaching influence upon our practical dealing with patients, and especially with convalescents. It has long been considered that those who have been, as the saying is, "pulled down" by illness, overwork or exhausting influences are more particularly liable to be attacked by tuberculosis. They are, in modern parlance, "fit soil" for the growth of the bacillus, and we are all accustomed to insist that until the health of such patients becomes re-established, they shall be carefully protected from exposure to infection by tuberculosis. According to the view so ably reinforced by Dr. Burton-Fanning we must go much further than this. Accepting the statement that some 40 per cent. of the population have a more or less marked tuberculous deposit hidden away somewhere or another within them, we may at once assume that anyone who has in previous years shown any "delicacy" is likely to be one of this tuberculous host, and that in dealing with such a patient everything must be avoided which might bring this latent tuberculosis into activity. This brings us to a point which is more particularly raised by Dr. Burton-Fanning, namely, the influence of overwork and anxiety in the etiology of consumption. Physical over-exertion he believes to be a most powerful determining cause of the breakdown of the system's limiting or protecting agencies against tuberculisation. In 10 per cent. of his cases he attributed the occurrence of manifest pulmonary tuberculosis to over-fatigue. Even a single excess in this respect may do the mischief; thus one of his patients was said never to have recovered from the exhaustion consequent on a whole day's bicycling in very hot weather and without previous preparation. Over-indulgence in tennis, dancing, climbing, and hunting have also seemed to be sufficient to bring into activity an unsuspected latent tuberculosis. In the management of convalescence from acknowledged tuberculosis, over-exertion is to be especially avoided. "Over and over again recrudescence of the malady has been brought about by a tiring journey, by too long a day of shopping or sight-seeing, by allowing the calls of family or society to interfere with the ordained hours of rest."

[1] Practitioner, March, 1902.

